# A novel approach to forecast global CO_2_ emission using Bat and Cuckoo optimization algorithms

**DOI:** 10.1016/j.mex.2020.100986

**Published:** 2020-07-09

**Authors:** Mojtaba Bahmani, Amin GhasemiNejad, Fateme Nazari Robati, Naeeme Amani Zarin

**Affiliations:** Department of Economics, Faculty of Management and Economics, Shahid Bahonar University of Kerman, Kerman, Iran

**Keywords:** Global climate changes, Meta heuristic method, Global warming

## Abstract

This paper presents the application of Bat and Cuckoo optimization algorithm methods to forecast Global CO_2_ emerged from energy consumption. The models are developed in two forms (linear and exponential) and used to estimate to develop Global CO2 emission model values based on the uses global oil, natural gas, coal, primary energy consumption. The available data are partly used for finding optimal, or near optimal values of weighting parameters (1980–2013) and partly for testing the models (2014–2018). The performance of methods is evaluated with mean squared error (MSE), root mean squared error (RMSE), Mean absolute error (MAE). According to the simulation results obtained, there is a good agreement between the results obtained from BA Global CO_2 emission models (BA-GCO_2) and COA Global CO_2 emission models (COA-GCO_2) but COA- exponential model outperformed the other models. The modeling approach recommended a helpful and reliable method for forecasting global climate changes and environmental decision making.

• The article provides a method for forecasting and climate policy decision making.
• The method presented in this article can be useful for experts, policy planners and researchers who study greenhouse gases.
• The analysis obtained herein by Metaheuristic Algorithms solver can serve as a standard benchmark for other researchers to compare their analysis of the other methods using this dataset.

## Graphical abstract

**Figure:**
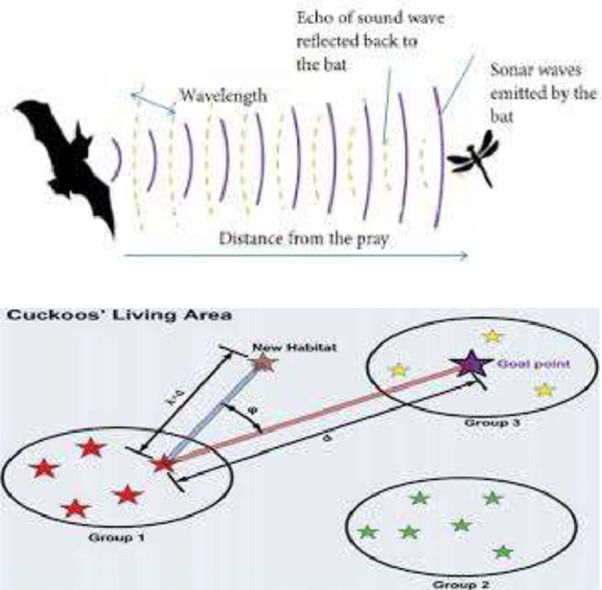

Specifications Table

**Table:** 

Subject Area	Environmental Science
More specific subject area	optimization methods, global climate changes
Method name	Bat optimization algorithm Cuckoo optimization algorithm
Name and reference of original method	[1] X.-S. Yang, "A new metaheuristic bat-inspired algorithm," in Nature inspired cooperative strategies for optimization (NICSO 2010), ed: Springer, 2010, pp. 65–74. [9] Yang, X.-S. and S. Deb. Cuckoo search via Lévy flights. in Nature & Biologically Inspired Computing, 2009. NaBIC 2009. World Congress on. 2009. IEEE [10] Rajabioun, R., Cuckoo optimization algorithm. Applied soft computing, 2011. 11(8): p. 5508–5518
Resource availability	12.The British Petroleum Company plc and BP Amoco plc. https://www.bp.com/content/dam/bp/business-sites/en/global/corporate/xlsx/energy economics/statistical-review/bp-stats-review-2019-all-data.xlsx

## Introduction

The use of non-classical methods in identifying models and predicting the behavior of complex systems has long been common in scientific circles. In many complex and especially non-linear systems, which modeling and subsequently their prediction and control through classical and analytical methods seems very difficult and sometimes impossible, non-classical methods that have features such as episteme-based intelligence and expertise are used. Nowadays, new methods have been developed for modeling and predicting various phenomena, in which evolutionary algorithms have a special place among them. This paper presents a combination of optimization algorithms (Bat and Cuckoo) and regression analysis (linear and exponential) to forecast Global CO2 emission. COA and BA are developed to Global CO_2_ emission values based on the natural gas, primary energy, global oil, coal consumption**.** The models have been developed in two forms (linear, exponential)

The linear form of the equation for Global CO_2_ emission model is written as follows:

(1) $$Y_{\text{linear}}=b_{1}Z_{1}+b_{2}Z_{2}+b_{3}Z_{3}+b_{4}Z_{4}+\;b_{5}$$

The exponential form of the equation for Global CO_2_ emission model is written as follows:

(2) $$Y_{exponential}=b_{1}Z_{1}^{c_{6}}+b_{2}Z_{2}^{c_{7}}+b_{3}Z_{3}^{c_{8}}+b_{4}Z_{4}^{c_{9}}+b_{5}$$

Where y represents the estimated Global CO_2_ emission and Z_i_ values represent the four independent variables used as the predictors of y (Z_1_ is global oil consumption, Z_2_ is natural gas consumption, Z_3_ is coal consumption and Z_4_ is primary energy consumption).

## Methods and material

### Bat algorithm

The Bats Algorithm (BA) developed by Yang (2008) is a population-based meta-heuristic algorithm, in which it then was extended in 2010 [1]. Sound position feature enables bats to obtain their prey. In this matter, the bats generate very loud sound pulses and receive the echoes of the sounds as they return from the surroundings. Here, the pulses contain several characteristics that depend on the bat's hunting strategy and the type of animal they intend to hunt. It should be mentioned bats could produce a three-dimensional space around themselves by delaying the reflection and detection of reflection, the time difference between the two ears, and the change in the loudness of the reflected sound. Moreover, they can distinguish the distance, direction, and even the velocity of their prey. The logic of this algorithm is that each virtual bat flies randomly at a velocity equal to ν_i_. Its position, i.e. *x_i_*, is the final solution. In this way, a bat varies the loudness (*A_i_*) and pulsation emission rate (*r_i_*) during a search to locate a prey. Besides, searching is also intensified using a local random walk. The selection of the best continues as long as one of the stop criteria is met. [2], [3], [4]

For simplicity, to create a BA, only some of the characteristics of bats are utilized as follows:

1. All bats employ the ability of sound position to detect the distance while they can distinguish between food and obstacles.
2. Although the loudness can be changed in different ways, it is assumed that the loudness can be varied from a large positive value of *A*_0_ to a small value of *A_min_*.
3. All bats randomly fly to find their prey at a velocity of *ν*_i_ in a position *x_i_* with a constant frequency *f_min_*, a variable wavelength *λ*, at a loudness *A*_0_

**Fig. 1:**
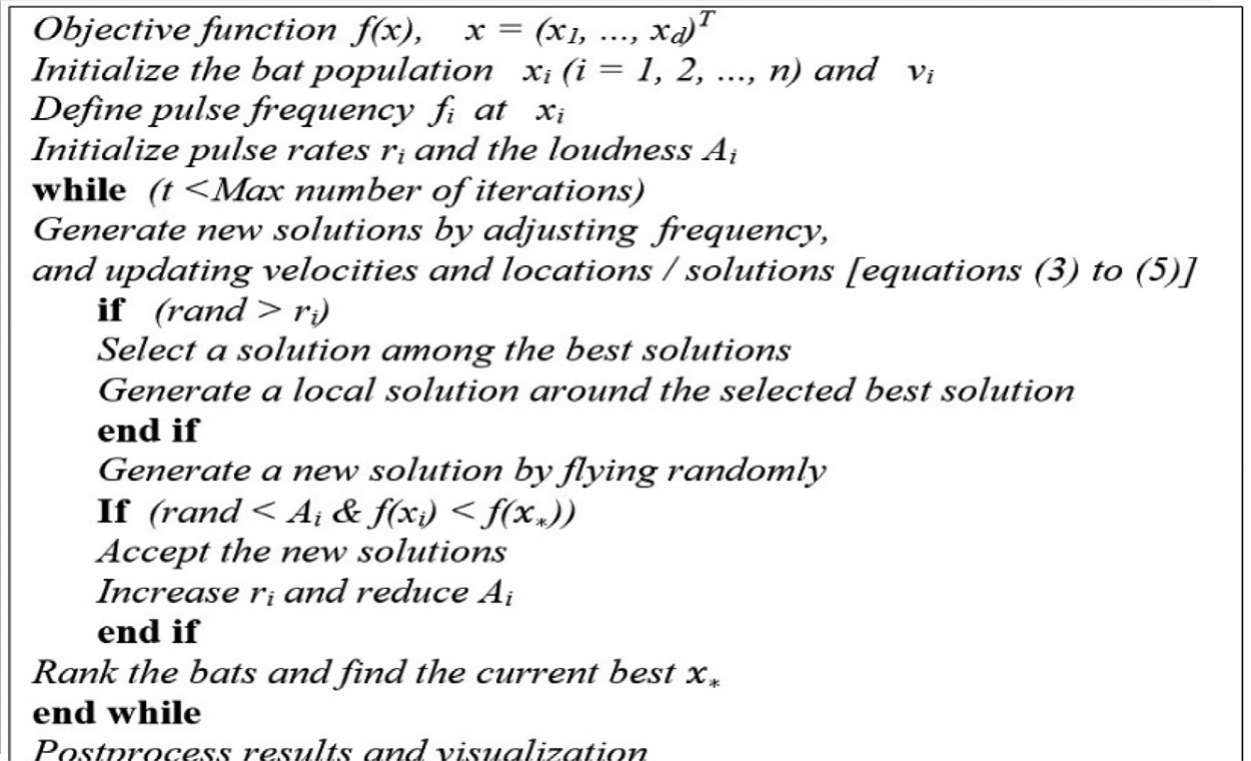
Pseudo code of the bat algorithm (BA).

In addition to the aforementioned simplification assumptions, other approximations have also been conducted in BA design such as *f* is usually in the range of [f_min_,  f_max_]. The amplitude of the wavelength should be chosen in accordance with the size of the problem being solved, and then it should be changed to a smaller size. In this algorithm, the position (one solution) and the velocity of each bat in the *t* *+* *1* step is measured as follows:

(3) $$f_{i}=\;f_{min}+\left(f_{max}-f_{min}\right)\beta$$

(4) $$\nu_{i}\left(t+1\right)=\nu_{i}\left(t\right)+\left(x_{i}\left(t+1\right)-x_{best}\right)f_{i}$$

(5) $$x_{i}\left(t+1\right)=x_{i}\left(t\right)+\nu_{i}\left(t\right)$$

where *β* ∈ [0, 1] is a uniform random vector and *x_best_* is the best global position among bats that are obtained up to now. The updating procedure of both position and velocity in bats contains similarities to the particle swarm optimization (PSO) algorithm, in which *f* controls particle velocity and swarm. Indeed, the BA can be well known as a combination of the PSO algorithm without taking advantage of the best local response of the *i-*particle, along with a local search based on the loudness and pulsation emission rate. [5,6]. Based on these approximations and idealization, the basic steps of the Bat Algorithm (BA) can be summarized as the pseudo code shown in Fig. 1.

**Fig. 2:**
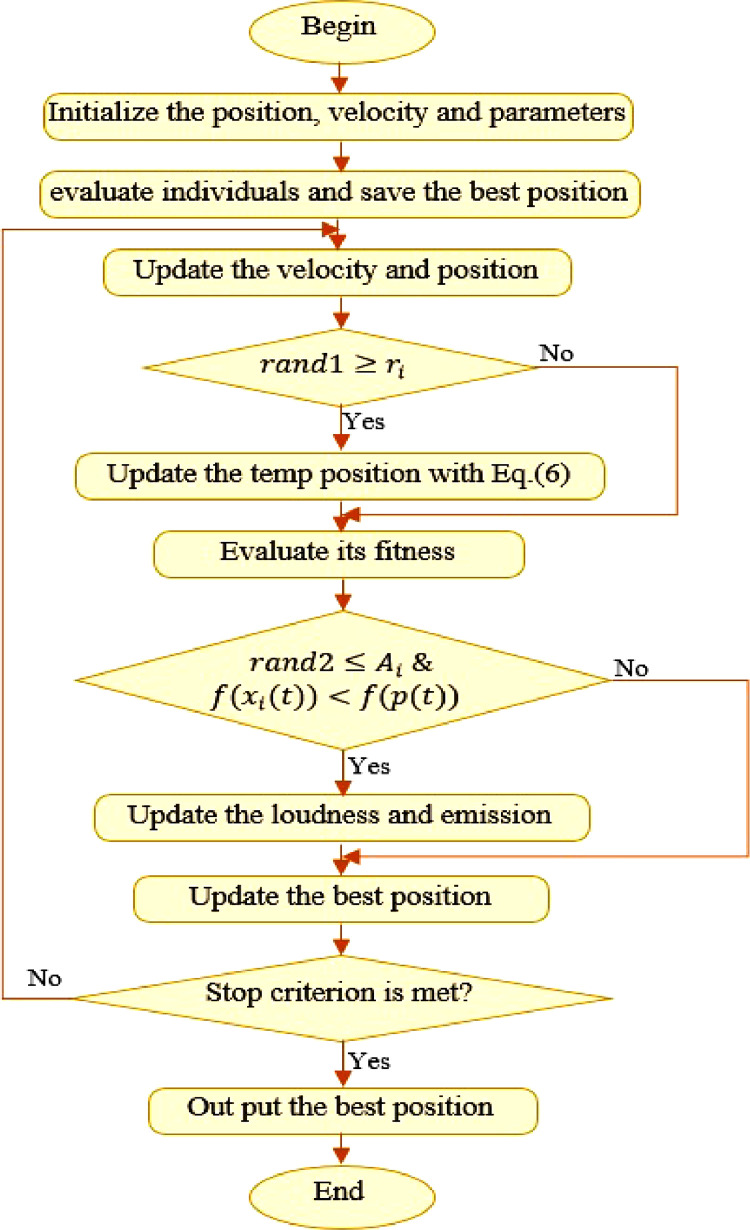
The flowchart of the standard bat algorithm (BA).

For the local search section, one solution is selected from the best current solutions, and then a new solution is locally generated for each bat using a random walk, i.e.

(6) $$x_{new}=\;x_{old}+\varepsilon A_{i}^{t}$$

where ε ∈ [0, 1] is a random value and *A^t^* t is the mean loudness of all bats at the time *t*. As a bat decreases its loudness (*A_i_*) when it detects a prey, and then increases its pulsation emission rate(*r_i_*), both *r_i_* and *A_i_* must be updated during the repetition process using the following relationships.

(7) $$A_{i}^{t+1}=\;\alpha A_{i}^{t}$$

(8) $$r_{i}^{t+1}=\;r_{i}^{0}\left[1-\exp\left(\gamma^{t}\right)\right]$$

where both α and γ are constant parameters and are considered at γ = α = 0.9 for simplicity. [7,8].Fig. 2 shows a flowchart of the bat algorithm.

### Cuckoo optimization algorithm

The cuckoo algorithm is a new method of deliberate global search inspired by the life of a bird called cuckoo. [9] Like other evolutionary algorithms, COA starts with an initial population. It is a population of cuckoos. These cuckoos have a number of eggs that will dump in some host birds’ nests. Some of these eggs, which are more similar to the host bird's eggs, have a better chance of growing into a mature cuckoo. Other eggs are identified and killed by the host bird. The number of grown-up eggs indicates the appropriateness of the nests in that area. The more eggs can survive in an area, the more profit (willingness) is ascribed to that area. Therefore, the situation in which more eggs survive will be the parameter that COA intends to optimize. Cuckoos search for the best area to maximize their eggs survival rate. Each cuckoo accidentally dumps eggs in the host bird's nest, which is within its egg-laying radius or ELR. In an optimization problem, each variable has an upper limit, var_hi_ and a lower limit var_low_, and each ELR can be defined using these limits. The ELR ratio for each cuckoo is determined as function (9).

(9) $$ELR=\;\alpha \times \frac{gn}{NI}\times \left(var_{hi}-var_{low}\right)$$

**Fig. 3:**
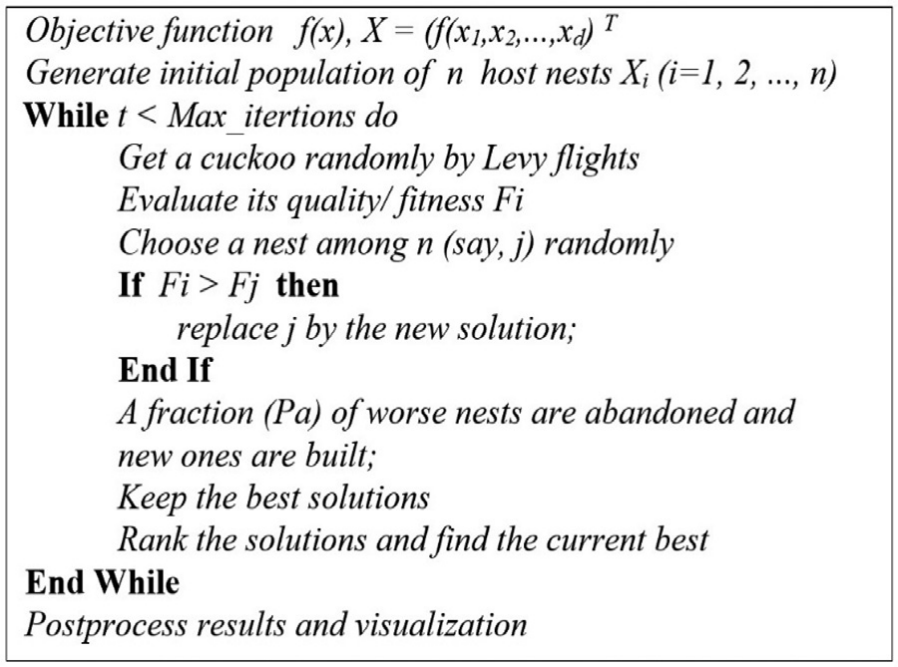
Pseudo code of the Cuckoo optimization Algorithm.

NI is the maximum number of iterations, *gn* is the current iteration, and α is the variable with which the maximum value of ELR is set. When all the cuckoos have laid their eggs, some of the eggs, which are not much similar to the host bird's eggs, are identified and thrown out of the nest [10]. Another interesting point about cuckoos is that only one egg can grow in each nest. The main steps of COA are presented in Fig. 3 as a pseudo-code.

**Fig. 4:**
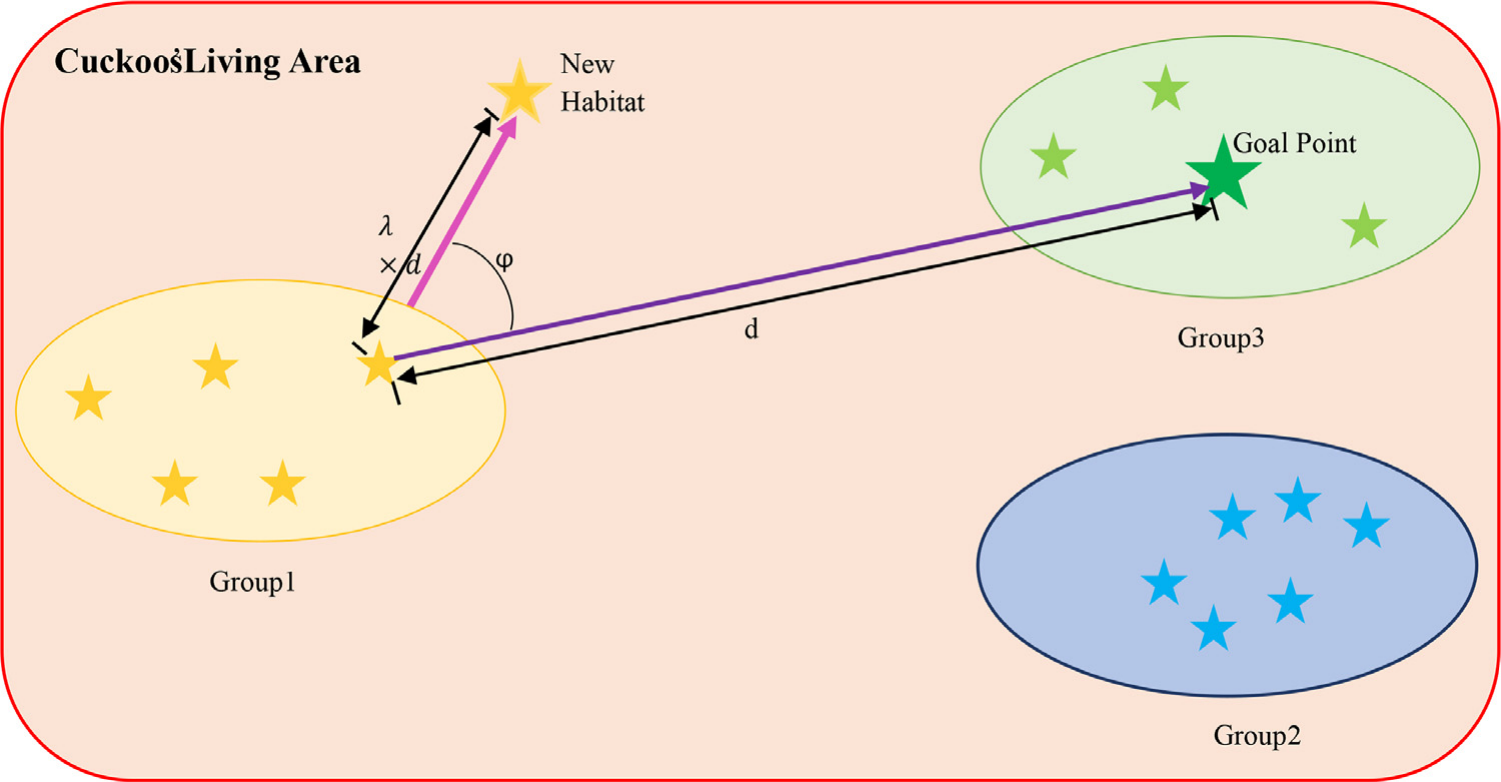
Migration behavior of the cuckoos.

When the chicks grow and mature, they live in their own environment and groups for a while, but when the time for egg-laying approaches, they migrate to better habitats where there are higher chances for the egg's survival. After the formation of cuckoo groups in different biological areas (problem search space), the group with the best position is selected as the target point for other cuckoos to immigrate. When adult cuckoos live in all over the environment, it is difficult to determine which cuckoo belongs to which group. To solve this problem, the cuckoos grouping is done by K-means clustering method. Now that cuckoo groups have been formed, their mean profit value is calculated to obtain the relative optimality of the group's habitat. Then the group with the highest mean profit value (optimization) is selected as the target group and other groups migrate toward it. While migrating toward the target point, the cuckoos do not travel all the way directly to the target location. They only travel part of the way and have a deviation along that path. You can clearly see this movement in Fig. 4.

When all cuckoos migrate to the target point and their new habitats are identified, each cuckoo lays a number of eggs. Depending on the number of eggs of each cuckoo, an ELR is determined for it and then egg-laying begins. Considering the fact that there is always a balance in the bird population in nature, a number like N_max_ controls and limits the maximum number of cuckoos that can live in an environment. After a few iterations, all the cuckoos reach an optimal point with the maximum similarity of the eggs to the host birds’ eggs, and the location with the maximum food resources. This habitat has the most overall profit and the least number of egg losses. The formula for the migration operator in the cuckoo optimization algorithm is function (10).

(10) $$X_{i}\left(k+1\right)=X_{i}\left(k\right)+F\times \left(X_{\text{GlobalBest}}-X_{i}\left(k\right)\right)$$

**Fig. 5:**
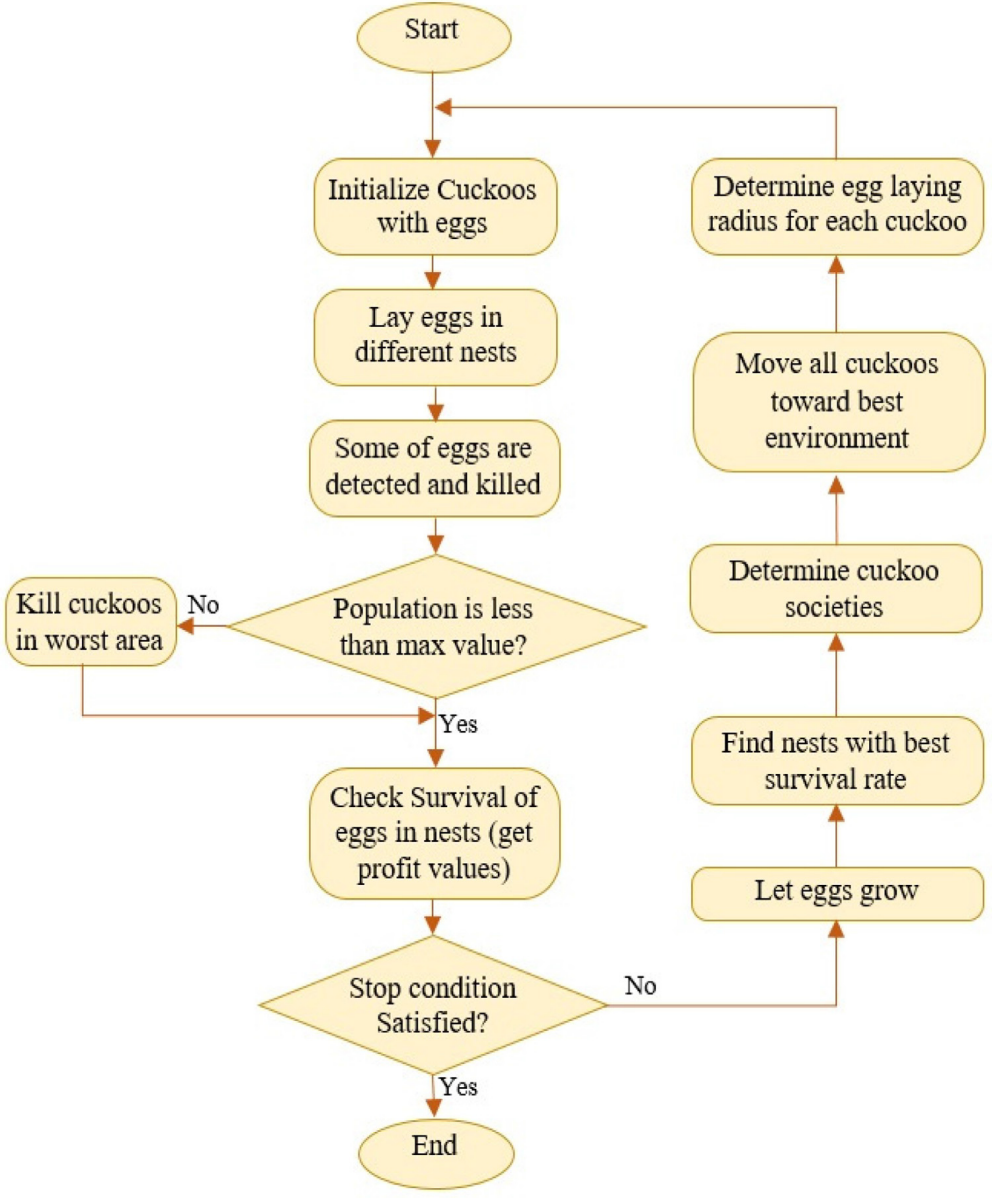
Flowchart of Cuckoo Optimization Algorithm.

Where *X_i_*(*k*) is the position of each cuckoo in iteration k, X_GlobalBest_ is the best optimal point in each group. Fig. 5 presents a flow diagram of bat algorithm.

### Optimization and method validation

Application of Bat algorithm (BA) together with Cuckoo Optimization Algorithm (COA) techniques to estimate the Global CO_2_ emission is the main objective of this paper**.**

The fitness function for selecting candidates for optimal coefficients is defined as:

(11) $$\text{Min}\;F\left(Z\right)=\sum_{j=1}^{k}{\left(Y_{\text{observed}}-Y_{\text{predicted}}\right)}^{2}$$

Where Y_observed_ and Y_predicted_ are the observed and predicted Global CO_2_ emission respectively, and k is the number of observations. [11]

In addition, error metrics MSE,^1^ MAE,^2^ RMSE^3^ were used to compare the performance of linear and exponential models to provide the best data analysis. These errors are specified in the forms of Eqs. (12)-(14)

(12) $$MSE=\frac{1}{n}\sum_{i=1}^{n}{\left(y_{\text{observed}}^{i}-y_{\text{predicted}}^{i}\right)}^{2}\;$$

(13) $$MAE=\frac{1}{n}\sum_{i=1}^{n}\;\left|y_{\text{observed}}^{i}-y_{\text{predicted}}^{i}\right|\;$$

(14) $$\text{RMSE}=\sqrt{\frac{1}{n}\sum_{i=1}^{n}{\left(y_{\text{observed}}^{i}-y_{\text{predicted}}^{i}\right)}^{2}\;}$$

Global CO_2_ emission from 1980 to 2018 is considered as a case in point in this paper. The available data is partly used for finding the optimal, or near optimal, values of the weighting parameters (1980–2013) and partly for validating the methods (2014–2018). [12]

Following steps are conducted for estimating Global CO_2_ emission between 1980 and 2018:

**Step 1:** natural gas, global oil, primary energy, coal consumption and Global CO_2_ emission need normalizing according to Eq. (15):

(15) $$Z_{N}=\left(Z_{R}-Z_{min}\right)/\left(Z_{max}-Z_{min}\right)$$

Z_N_: Normalized value, Z_R_: The value to be normalized, Z_min_: The minimum value in all the values for related variable, Z_max_: The maximum value in all the values for related variable. The Z_min_ and Z_max_ values for each variable are selected between 1980 and 2013 and are shown in Table 1.

**Table 1:** Values for normalization.

	Z_min_	Z_max_
global oil consumption (Million tonnes)	2841.4	4350.3
natural gas (Billion cubic meters)	1224.2	2897.5
coal (Million tonnes oil equivalent)	1793.3	3867
primary energy consumption (Million tonnes oil equivalent)	6507.4	12,819.4
Global CO_2_ emission (Million tonnes of carbon dioxide)	17,976.7	32,799.9

**Step 2:** The proposed methods are used in order to determine corresponding weighting factors (C_i_) for each model according to the lowest objective functions. [11]

**Step 3:** The best results of step 2 for each model and less average relative errors in the testing period are chosen (i.e., the related data from 2014 to 2018). The best-obtained weighting factors by COA and BA for linear and exponential models are shown in Table 2.

**Table 2:** The best-obtained weighting factors by COA and BA optimization algorithms.

Algorithm Model	C1	C2	C3	C4	C5	C6	C7	C8	C9
$Y_{\mathrm{BA}-\mathrm{GC}{O_{2}}_{\mathrm{linear}}}$	0.2760	0.1797	0.2921	0.0129	0.1217	–	–	–	–
$Y_{\mathrm{BA}-\mathrm{GC}{O_{2}}_{\mathrm{exponential}}}$	0.0904	0.0144	0.1053	0.0136	0.4437	0.1246	0.2689	0.0034	0.4270
$Y_{\mathrm{COA}-\mathrm{GC}{O_{2}}_{\mathrm{linear}}}$	0.0387	0.2848	0.433	0.0002	0.1228	–	–	–	–
$Y_{\mathrm{COA}-\mathrm{GC}{O_{2}}_{\mathrm{exponential}}}$	0.1997	0.0213	0.1948	0.0074	0.4231	0.0803	0.7234	0.3010	0.6827

The obtained values of MSE, RMSE and MAE for both methods are presented in Table 3.

**Table 3:** Comparison of the Performance evaluation of linear, exponential COA and BA optimization algorithms performance criteria.

Algorithm Model	MSE	RMSE	MAE
$Y_{\text{BA}-\text{GC}{O_{2}}_{\text{linear}}}$	0.0995	0.3155	0.2595
$Y_{\text{BA}-\text{GC}{O_{2}}_{\text{exponential}}}$	0.0898	0.2996	0.2322
$Y_{\text{COA}-\text{GC}{O_{2}}_{\text{linear}}}$	0.0937	0.3061	0.2469
$Y_{\text{COA}-\text{GC}{O_{2}}_{\text{exponential}}}$	0.0404	0.2011	0.1865

It can be seen that there is a good agreement between the results obtained from BA Global CO_2_ emission models ($\text{BA}-GCO_{2}$) and COA Global CO_2_ emission models ($\text{COA}-GCO_{2}$) but COA- exponential model outperformed the other models.

Tables 4 and 5 for the modeling and the testing data show the performance of the Bat and cuckoo optimization algorithm for all models. The findings proved that the recommended exponential model was more appropriate tool for forecasting Global co2 emission.

**Table 4:** Comparison of the $\text{BA}-GCO_{2}$ estimation models for Global CO_2_ emission in testing period (2014–2018).

Years	Actual data (Mboe)a	$\text{BA}-\text{GC}{O_{2}}_{\text{linear}}$	Relative error%	$\text{BA}-\text{GC}{O_{2}}_{\text{exponential}}$	Relative error%
2014	32,844.80	29,891.78392	−8.990	30,157.64064	−1.282399386
2015	32,804.40	30,292.49698	−7.6572	30,213.50655	−1.120979953
2016	32,913.50	30,766.9375	−6.5218	30,325.93366	−1.407552133
2017	33,242.50	31,364.16542	−5.6504	30,507.50182	−2.354015662
2018	33,890.80	32,465.33439	−4.2060	30,824.42145	−4.194706935
Average	–	–	6.6052	–	2.7193

a Actual data is in million tonnes of carbon dioxide.

**Table 5:** Comparison of the $\text{COA}-GCO_{2}$ estimation models for Global CO_2_ emission in testing period (2014–2018).

years	Actual data (Mboe)a	$\text{COA}-\text{GC}{O_{2}}_{\text{linear}}$	Relative error%	$\text{COA}-\text{GC}{O_{2}}_{\text{exponential}}$	Relative error%
2014	32,844.80	31,381.78352	−4.454332133	33,119.62228	0.836729968
2015	32,804.40	31,639.44806	−3.551206357	33,149.3367	1.051495231
2016	32,913.50	31,916.43871	−3.029338391	33,317.10684	1.226265336
2017	33,242.50	32,157.61566	−3.263546176	33,679.26364	1.313871227
2018	33,890.80	32,469.1793	−4.194709769	34,273.20273	1.128337874
Average	–	–	3.6986	–	1.11134

a Actual data is in million tonnes of carbon dioxide.

## Conclusion

The point of this study is to demonstrate to the authorities the significance of utilizing option estimating strategies. Therefore, in this study COA (cuckoo optimization algorithm) has been successfully used to estimate Global CO_2_ emission based on global oil, natural gas, coal, primary energy consumption. To make a conclusion, this paper presented helpful suggestions and novel insights for experts and policy planners. It recommended a strong tool for developing energy programs. Forecasting Global CO_2_ emission can as well be studied by neural networks or other new metaheuristics including harmony search, simulated annealing, etc. The results of different methods may be compared with COA method.

## Declaration of Competing Interest

The authors declare that they have no known competing financial interests or personal relationships that could have appeared to influence the work reported in this paper.

## Footnotes

1 Mean squared error.

2 Mean absolute error.

3 Root mean squared error.
